# Towards continuous industrial bioprocessing with solventogenic and acetogenic clostridia: challenges, progress and perspectives

**DOI:** 10.1007/s10295-020-02296-2

**Published:** 2020-09-07

**Authors:** Charlotte Anne Vees, Christian Simon Neuendorf, Stefan Pflügl

**Affiliations:** Institute of Chemical, Environmental and Bioscience Engineering, Research Area Biochemical Engineering, Technische Universität Wien, Gumpendorfer Straße 1a, 1060 Vienna, Austria

**Keywords:** Cell retention and immobilization, Systems biology and genome-scale metabolic models, Complex and renewable feedstocks, Gas fermentation, Integrated product recovery

## Abstract

The sustainable production of solvents from above ground carbon is highly desired. Several clostridia naturally produce solvents and use a variety of renewable and waste-derived substrates such as lignocellulosic biomass and gas mixtures containing H_2_/CO_2_ or CO. To enable economically viable production of solvents and biofuels such as ethanol and butanol, the high productivity of continuous bioprocesses is needed. While the first industrial-scale gas fermentation facility operates continuously, the acetone–butanol–ethanol (ABE) fermentation is traditionally operated in batch mode. This review highlights the benefits of continuous bioprocessing for solvent production and underlines the progress made towards its establishment. Based on metabolic capabilities of solvent producing clostridia, we discuss recent advances in systems-level understanding and genome engineering. On the process side, we focus on innovative fermentation methods and integrated product recovery to overcome the limitations of the classical one-stage chemostat and give an overview of the current industrial bioproduction of solvents.

## Introduction

The Paris Agreement adopted in 2016 displays an international effort to reduce carbon emissions and promotes the development of new sustainable processes for fuel and chemical production using “above ground” carbon as feedstocks [149]. To implement sustainable and economically viable processes towards the establishment of a circular bioeconomy, the use of cheap and abundant carbon sources such as municipal solid waste, lignocellulosic biomass and steel mill exhaust gas must be favored over expensive and edible carbon sources like starch [149, 292]. Solventogenic clostridia can grow on a variety of hexose and pentose sugars and produce relevant solvents such as ethanol, butanol and acetone. The Weizmann process was implemented more than a hundred years ago [255], making solventogenic clostridia long-known production hosts of the industrial biotechnology. Acetogens can grow on mixtures of CO, CO_2_ and H_2_ which can be obtained directly from furnaces of steel mills or through the gasification of various carbon-rich waste streams and lignocellulosic biomass [170]. The product spectrum strongly depends on the acetogenic strain and includes the commodity chemicals acetate, ethanol and butanol [19, 59]. To enable the commercialization of bioprocess for the production of bulk chemicals like solvents, an estimated product titer of 50 g L^−1^, the productivity of 3 g L^−1^ h^−1^ and yield not less than 80% of the theoretical yield have to be reached [302]. Continuous bioprocessing offers a mean to reach the demanded high productivity [21, 208, 315].

Biofuels such as butanol and ethanol are needed in high quantities and their market shows a steady growth [164, 261, 270, 275]. While ethanol is already used worldwide for biofuel applications, a 50% higher energy density, lower vapor pressure, lower water absorption, lower corrosivity, better blending abilities and the possible use in unmodified combustion engines and existing infrastructure make butanol a promising alternative [41, 58, 161, 211]. To penetrate the biofuel market, butanol production has to compete with the performance of ethanol-producing bioprocesses [95]. Continuous ethanol production reached productivities of ~ 10 g L^−1^ h^−1^, yields of up to 0.46 g ethanol per g of pentose or hexose and concentrations of ~ 100 g L^−1^ [229]. The first commercial scale gas fermenting facility for ethanol production started operation in 2018 and runs in a fully continuous manner with a comparable productivity [149, 284]. The acetone–butanol–ethanol (ABE) fermentation, however, is classically operated in batch mode and the switch to a continuous bioprocess proves challenging [95, 164, 219]. Continuous high cell density cultivations of solventogenic clostridia have already reached butanol productivities of about 10 g L^−1^ h^−1^ [125, 187]. Due to the high toxicity of butanol, titers are typically limited to values below 20 g L^−1^ [79, 133]. Integrated product recovery methods display a meaningful way to compensate for the low product titers and to alleviate product toxicity [82].

In this review, we show the progress made towards the continuous production of solvents with solventogenic and acetogenic clostridia. With the objective of a holistic process design, the first part of the review focuses on the production hosts where we highlight metabolic capabilities and relevant phenotypical properties of clostridia. The recent developments in systems biology and genetic engineering tools increase microbial understanding and enable better strain design. Regarding the implementation of a sustainable and economical process, we give a short overview of the most promising alternative feedstocks. By building the bridge to the current advances of fermentation methods and add-ons used for solvent production, we discuss the challenges and opportunities of continuous fermentation and outline the current situation of industrial bioprocessing for solvent production. Finally, we tie up the threads for the successful industrial implementation of continuous solvent production by emphasizing the importance to combine strain engineering with innovative fermentation methods along with the need for further improvement of monitoring and control strategies for these processes.

## Solventogenic and acetogenic clostridia

Solventogenic clostridia have been a part of industrial biotechnology as production hosts of solvents for more than a century [255]. While the research focused on *Clostridium acetobutylicum*, the model organism of the ABE fermentation, further clostridia including *C. beijerinckii*, *C. saccharoperbutylacetonicum* and *C. saccharobutylicum* were investigated for their high butanol production activity [58, 143, 166]. With the isolation of *C. ljungdahlii,* acetogenic bacteria (acetogens) have also become interesting hosts for industrial solvent production. This organism was first studied for its ability to form ethanol from gasified coal and is today one of the model acetogens [258, 315]. Acetogens are more relevant than ever as they can utilize the greenhouse gases CO and CO_2_ as inorganic carbon sources, making them applicable for carbon capture and valorization technologies [61, 149]. Acetogens form a metabolically, ecologically, and phylogenetically diverse group [256]. Several acetogenic clostridia such as *C. ljungdahlii, C. autoethanogenum* and *C. carboxidivorans* are investigated for solvent production [19, 59]. Non-clostridial acetogens such as *Acetobacterium woodii* and *Eubacterium limosum* are also investigated and modified for the production of bulk chemicals [108, 128, 276]. The most common solventogenic and acetogenic clostridia investigated for industrial application are summarized in Table 1.

**Table 1:** Overview of industrially relevant solventogenic and acetogenic clostridia

Strain	Growth on sole carbon source:					Native fermentation products	References
	H_2_/CO_2_	CO/CO_2_	C6 sugars	C5 sugars	Glycerol		
*C. acetobutylicum*	–	–	X	X	(X)	Acetate, acetone, butanol, butyrate, ethanol, H_2_, CO_2_	[143, 157]
*C. beijerinckii*	–	–	X	X	(X)	Acetate, acetone, butanol, butyrate, ethanol isopropanol^a^, H_2_, CO_2_	[143, 197, 279]
*C. saccharoperbutylacetonicum*	–	–	X	X	–	Acetate, acetone, butanol, butyrate, ethanol, H_2_, CO_2_	[143]
*C. saccharobutylicum*	–	–	X	X	–	Acetate, acetone, butanol, butyrate, ethanol, H_2_, CO_2_	[143]
*C. pasteurianum*	–	–	X	–	X	Acetate, Butanol, butyrate, ethanol, lactate, 1,3-propanediol, H_2_, CO_2_	[223]
*C. ljungdahlii*	X	X	X	X	–	Acetate, 2,3-butanediol, ethanol, lactate, CO_2_	[148, 286]
*C. carboxidivorans*	X	X	X	X	X	Acetate, ethanol, butanol, butyrate, hexanoate, hexanol, CO_2_	[80, 172]
*C. ragsdalei*	X	X	X	X	–	Acetate, ethanol, 2,3-butanediol, lactate, CO_2_	[115, 148]
*C. drakei*	X	X	X	X	X	Acetate, ethanol, butanol, butyrate, CO_2_	[172]
*C. autoethanogenum*	X	X	X	X	–	Acetate, ethanol, 2,3-butanediol, lactate, CO_2_	[3, 148, 192]
*M. thermoacetica*	X	X	X	X	–	Acetate, CO_2_	[56]

X: growth, (X): weak growth [143], –: no growth

^a^Only some strains like *C. beijerinckii *DSM 6423 synthesize isopropanol [279]

### Metabolic modules

**Fig. 1:**
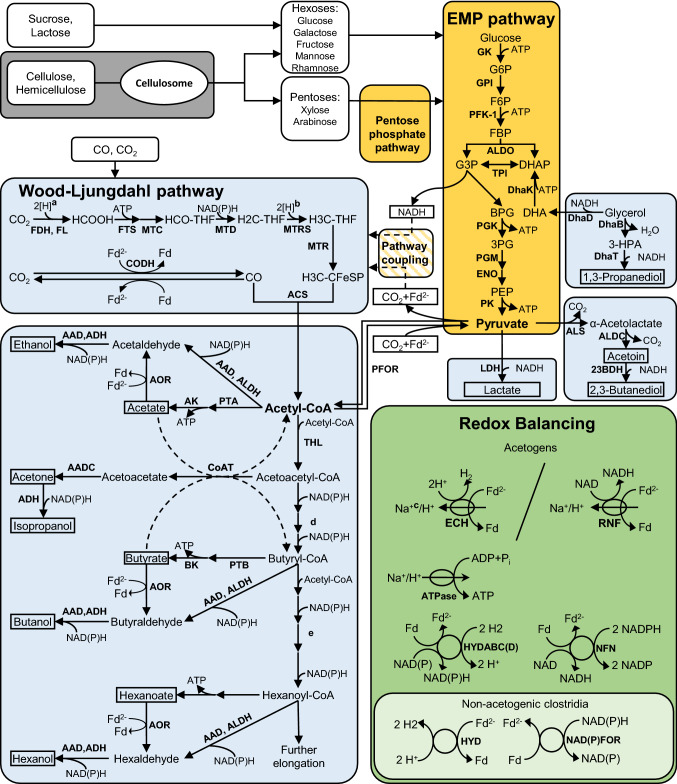
Schematic of the metabolism of acetogenic and solventogenic clostridia. Oxidative metabolic modules for the generation of reduction equivalents and intermediates are depicted in yellow. Reductive metabolic modules consuming reduction equivalents and synthesizing products are displayed in light blue. Redox balancing modules for the balancing of formed and consumed reduction equivalents are marked in green. Products of reductive metabolic modules are framed by black boxes. **a** Reduction of CO_2_ to formate can use H_2_, Fd^2−^, NADPH or even 0.5 Fd + 0.5 NADPH; **b** NADH is used for the reduction of H2C-THF to H3C-THF in the non-clostridial acetogen *Acetobacterium woodii*. In *C. autoethanogenum*, 2 NADH are most likely used to reduce Fd and H2C-THF in an electron bifurcating reaction [300]. **c** The translocation of Na^+^ by Ech in some species is likely but experimental evidence is missing [258]. **d** Subsequent steps for the reduction of acetoacetyl-CoA to butyryl-CoA are catalyzed by 3-hydroxyacyl-CoA dehydrogenase, crotonase and acyl-CoA dehydrogenase.** e** Subsequent steps for the reduction of butyryl-CoA to hexanoyl-CoA are catalyzed by thiolase, 3-hydroxyacyl-CoA dehydrogenase, crotonase and acyl-CoA dehydrogenase. *23BDH* 2,3-butanediol dehydrogenase; *3-HPA* 3-hydroxypropionaldehyde; *3PG* glycerate 3-phosphate; *AAD* alcohol/aldehyde dehydrogenase; *AADC* acetoacetate decarboxylase; *ACS* acetyl-CoA synthase; *ADH* alcohol dehydrogenase; *AK* acetate kinase; *ALDC* acetolactate decarboxylase; *ALDH* aldehyde dehydrogenase; *ALDO* fructose biphosphate aldolase; *ALS* acetolactate synthase; *BK* butyrate kinase; *BPG* 1,3-bisphosphoglycerate; *CoAT* CoA transferase; *CFeSP* corrinoid iron–sulfur protein; *DHA* dihydroxyacetone; *DhaB* glycerol dehydratase; *DhaD* glycerol dehydratase; *DhaK* DHA kinase; *DHAP* dihydroxyacetone phosphate; *DhaT* 1,3-propanediol oxidoreductase; *ECH* energy-converting hydrogenase complex; *ENO* enolase; *F6P* fructose 6-phosphate; *FBP* fructose 1,6-bisphosphate; *Fd* ferredoxin; *FDH* formate dehydrogenase; *FL* formate-H_2_ lyase; *FTS* formyl-THF synthase; *G3P* glyceraldehyde 3-phosphate; *G6P* glucose 6-phosphate, *GAPDH* glyceraldehyde phosphate dehydrogenase; *GK* hexokinase; *GPI* phosphoglucose isomerase; *HYD* hydrogenase; *HYDABC(D)* electron-bifurcating hydrogenase; *LDH* lactate dehydrogenase; *MTC* methenyl-THF cyclohydrolase; *MTD* methylene-THF dehydrogenase; *MTR* methyl transferase; *MTRS* methylene-THF reductase; *NAD(P)FOR* NAD(P)H:Ferredoxin oxidoreductase; *NFN* electron-bifurcating transhydrogenase; *PFK-1* phosphofructokinase; *PFOR* pyruvate:ferredoxin oxidoreductase; *PGK* phosphoglycerate kinase; *PGM* phosphoglycerate mutase; *PEP* phosphoenolpyruvate; *PK* pyruvate kinase; *PTA* phosphotransacetylase; *PTB* phosphotransbutyrylase; *RNF* Rnf complex; *TPI* triosephosphate isomerase

The metabolism of solventogenic and acetogenic clostridia can be subdivided into metabolic modules (see Fig. 1). Oxidative metabolic modules break down heterotrophic carbon sources to the intermediates acetyl-CoA and pyruvate and generate reduction equivalents. Additional reduction equivalents can be obtained from CO and H_2_ oxidation. Acetogens possess the Wood–Ljungdahl pathway, a reductive metabolic module that uses reduction equivalents to fixate CO_2_ and to form additional acetyl-CoA [259]. Further reductive modules use reduction equivalents to convert intermediates to products such as butanol, ethanol, acetone and 2,3-butanediol (2,3-BDO) [69, 148, 245]. Balancing modules match the generated and consumed reduction equivalents.

Carbohydrates display a valuable carbon source for clostridia. Complex feedstocks such as lignocellulose may be used directly when the organisms are able to degrade it to fermentable sugars. Cellulolytic clostridia like *C. thermocellum* produce enzymatic complexes called cellulosomes for this task and are reviewed in detail elsewhere [194, 321]. Released or directly fed carbohydrates are degraded for energy and reduction equivalent generation. The interlinked Embden–Meyerhof–Parnas (EMP) and pentose phosphate pathways (PPP) are the oxidative metabolic modules responsible for the degradation of hexoses and pentoses, respectively [219]. Finally, pyruvate is formed and may be used for acetyl-CoA formation releasing CO_2_ and generating additional reduction equivalents.

Acetogens can generate acetyl-CoA via the Wood–Ljungdahl pathway (WLP). There are several reviews recommended for further reading [20, 57, 258, 259]. The WLP is a reductive module that can use reduction equivalents generated from oxidative modules (EMP and PPP) or from the oxidation of CO or H_2_ [20, 259]. CO_2_ is stepwise reduced to a methyl-group in the Eastern branch of the WLP. The Western branch serves to provide a carbonyl group either directly from CO or from the reduction of CO_2_. Finally, the methyl-group and a carbonyl-group are combined with coenzyme A (HS-CoA) to form acetyl-CoA [57].

The growth of acetogenic and solventogenic clostridia in batch cultivations can be divided into two phases (‘biphasic’ fermentation): First, produced coenzyme A-bound acids (acetyl-CoA, butyryl-CoA, hexanoyl-CoA) can be released enabling ATP generation and fast growth, leading to the overall production of acids. This growth phase is referred to as acidogenesis [124]. In a second growth phase, the accumulated acids are taken up and converted to alcohols by reductive modules. Due to the accumulation of solvents, this growth phase is called solventogenesis [247]. In solventogenic clostridia, coenzyme A bound acids are reduced to their respective aldehyde by alcohol/aldehyde dehydrogenases (AADs) or aldehyde dehydrogenases (ADH) [38, 317]. Several acetogenic clostridia harbor aldehyde oxidoreductases (AORs) for the direct conversion of carboxylic acids to aldehydes without prior activation [48, 80, 120, 247]. AORs were shown to guide the ethanol formation during autotrophic growth of *Clostridium autoethanogenum* [169]. However, the direct reduction of acetic acid to acetaldehyde is thermodynamically unfavorable under standard conditions (1 M concentration of acetic acid and acetaldehyde at pH 7) and is facilitated by a low intracellular pH value [198].

Stoichiometric imbalances of reduction equivalents are resolved by redox balance modules: acetogens possess a membrane-bound trans-hydrogenase (Ech or Rnf complex) that transfers electrons from electron carriers with low redox potential (Fd^2−^) to electron carriers with a higher redox potential (NAD/NADH, H_2_) and couples the transfer with the translocation of Na^+^ or H^+^ out of the cell [258]. The generated chemiosmotic gradient can be used for energy generation by a membrane-bound ATPase. Electron bifurcating hydrogenases like HydABCD are essential for the supply of reduced ferredoxin during growth on mixtures of H_2_ and CO_2_ and may also serve for redox balancing during heterotrophic growth [258, 316]. During acidogenic growth, solventogenic clostridia like *C. acetobutylicum* balance surplus NADH by forming H_2_ [218].

### Parameters and conditions promoting the solvent formation

Overall, the pH value, the acid concentration, and the degree of reduction of the substrate influence the metabolism and the formed products of acetogens and clostridia. These parameters can be used to steer the cultivation towards solventogenesis. During a continuous, phosphate-limited cultivation of *C. acetobutylicum* ATCC824, a change from acidogenic to solventogenic metabolism could be directed by solely changing the external pH from 5.7 to 4.5 [97, 126]. A two-stage continuous cultivation of *C. ljungdahlii* also allowed to control acidogenesis and solventogenesis using the pH setpoint [247]. Similarly, solventogenesis was induced during batch cultivation of the acetogen *C. aceticum* by shifting the pH-value from 8.0 to 6.9 [10]. Interestingly, the pH value was also suggested to favor alcohol formation reactions and to hamper the formation of longer fatty acids like hexanoic acid during the cultivation of acetogenic clostridia [41]. Supplementing a batch culture of *C. beijerinckii* NCIMB 8052 with acetate, butyrate or both led to an earlier onset of solventogenesis and to higher final butanol titers, highlighting the role of acid concentration in switching to solventogenesis [109, 313]. The supply of reduction equivalents during heterotrophic cultivation can be increased by changing the carbon source. Replacing glucose with glycerol for the cultivation of *C. pasteurianum* shifted the product spectrum from acids to solvents [46]. During continuous cultivation of the acetogen *C. autoethanogenum*, increasing the ratio of H_2_ to inorganic carbon in the feed gas led to an increased ratio of ethanol to acetate, showing that the amount of reduction equivalents supplied from the substrate influences solvent formation [299].

An interesting feature of some acetogens is the ability to use gaseous substrates and organic carbon sources like carbohydrates simultaneously. This ability is referred to as anaerobic, non-photosynthetic (ANP) mixotrophy [134]. By providing additional reduction equivalents via CO or H_2_ oxidation, the theoretical butanol yield on glucose is increased from 0.97 mol mol^−1^ to 1.33 mol mol^−1^ [76]. Advantages and applications of ANP mixotrophy are further discussed elsewhere [61, 76, 78, 192].

### Strain stability and changes in strain performance

Aside from parameters that support solventogenic growth behavior, influences on the cellular performance and viability have to be considered: solventogenic clostridia may partially or completely lose their ability to produce solvents from acids during continuous cultivation or repeated batch cultivation [141]. This phenotypical phenomenon called strain degeneration has various causes. In *C. acetobutylicum* ATCC 824, degeneration is caused by the loss of the mega plasmid pSol carrying the genes for solvent formation [45]. In case of the degenerated strain *C. beijerinckii* DG 8052, the ability to form solvents was lost without a genetic change and could be restored by addition of CaCO_3_ [131]. Even phage infection caused strain degeneration during the industrial cultivation of *C. madisonii* [132]. Interestingly, a degeneration-resistant strain of *C. beijerinckii* NCIMB 8052 was isolated as early as 1993 [142]. Degeneration has, to the best of our knowledge, not been observed for an acetogen yet.

During the so-called acid crash, the fast accumulation of acids causes the cultivation to end before switching to the solventogenic phase [16, 80]. The acid crash in *C. acetobutylicum* was shown to be caused by formic acid accumulation to concentrations of ~ 1 mM [311]. In case of the acetogen *C. carboxidivorans* P7, an acid crash was caused by the fast accumulation of acetic acid at high cultivation temperatures (37 °C) [245].

The solvents produced are toxic to the culture: the growth of the *C. acetobutylicum* ATCC 824 wild type was inhibited by 50% when butanol, ethanol and acetone were added in concentrations of 7–13 g L^−1^, 40 g L^−1^ and 40 g L^−1^, respectively. 20 g L^−1^ butanol inhibited growth completely [133]. Growth of *C. carboxidivorans* cultures with CO as the sole carbon source was inhibited to 50% or even completely by 14.5 and 20 g L^−1^ butanol, respectively. Tolerance against ethanol was significantly higher: 35 g L^−1^ ethanol inhibited growth to 50% [79].

The onset of solventogenesis is seen as a survival strategy for dealing with the rising acid concentration during batch cultivation. Sporulation is a second survival strategy of clostridia [326]. Both sporulation and the metabolic switch from acidogenesis to solventogenesis are coordinated by the master regulator Spo0A in *C. beijerinckii* NCIMB 8052 [246]. However, the coordination of both events seems to differ between clostridial strains [219] and is not completely resolved to date [166].

While problems with sporulation have been reported for the acid-producing strain *C. kluyveri* [89], sporulation so far has not been identified as a problem regarding acetogenic clostridia because *C. ljungdahlii* and *C. autoethanogenum* were found to rarely sporulate [3, 286].

## Systems biology and genetic engineering

The characterization of the metabolism of clostridial species and its regulation are the basis of metabolic engineering approaches on the way to high-performance strains for highly efficient industrial solvent production [333]. By applying omics technologies and metabolic modelling, our understanding of production hosts on the systems level is improved and can guide the rational strain design [40, 326, 333].

Genome-scale metabolic (GSM) models allow to describe the metabolic capabilities of different species [51]. GSM models have been developed for solventogenic clostridia such as *C. acetobutylicum* ATCC 824 and *C. beijerinckii* NCIMB 8052, cellulolytic clostridia such as *C. cellulolyticum* and *C. thermocellum* and several acetogenic clostridia such as *C. ljungdahlii*, *M. thermoacetica*, *C. autoethanogenum* and *C. drakei* (see Table 2).

**Table 2:** Summary of genome-scale metabolic models for *Clostridium* spp.

Organism	Acetogen	Metabolic model			References
		Genes	Reaction	Metabolites	
*C. acetobutylicum* ATCC 824	N	432	502	479	iJL432 [157]
*C. acetobutylicum* ATCC 824	N	473	522	422	[262]
*C. acetobutylicum* ATCC 824	N	700	709	679	iFS700 [252]
*C. acetobutylicum* ATCC 824	N	490	794	707	iCac490 [196]
*C. acetobutylicum* DSM 792	N	N/A	592	444	[309]
*C. acetobutylicum* ATCC 824	N	802	1462	1137	iCac802 [50]
*C. acetobutylicum* ATCC 824	N	967	1231	1058	iCac967 [332]
*C. beijerinckii* NCIMB 8052	N	925	938	881	iCM925 [197]
*C. butyricum* IBUN 13A	N	641	891	701	iCbu641 [263]
*C. cellulolyticum* H10	N	431	621	603	iFS431 [253]
*C. kluyveri*	N	708	994	804	iCKL708 [342]
*C. thermocellum* ATCC 27405	N	432	577	525	iSR432 [250]
*C. thermocellum* DSM 1313	N	601	872	904	iAT601 [290]
*C. thermocellum* ATCC 27405	N	446	637	598	iCth446 [49]
*C. autoethanogenum*	Y	805	1002	1075	[189]
*C. autoethanogenum*	Y	786	1109	1097	iCLAU786 [300]
*C. autoethanogenum*	Y	699	755	772	MetaCLAU [216]
*C. drakei*	Y	771	922	854	iSL771 [271]
*C. ljungdahlii*	Y	637	785	698	iHN637 [206]
*C. ljungdahlii*	Y	680	809	718	iJL680^a^ [177]
*M. thermoacetica*	Y	558	705	698	iAI558 [119]

^a^iJL680 is the GSM model that serves as the basis for the ME-model iJL965-ME. iJL965-ME extends iJL680 by adding 196 protein-coding open reading frames (ORFs), 89 RNA genes, 576 transcription units, 19 types of rRNA modifications, 17 types of tRNA modifications, 735 protein complexes with updated stoichiometry, 219 modified protein complexes and 134 translocated proteins

Grouping the metabolism as metabolic modules allows to compare the abilities of different organisms. Interestingly, fragments of modules might also be included in strains that cannot express an entire module functionally. As an example, several GSM models of clostridia contain the carbon monoxide dehydrogenase (CODH) reaction but only acetogens such as *C. ljungdahlii* harbor the full WLP. A common clostridial ancestor potentially had a functional WLP [51]. The modularity of the metabolism is an impetus for researchers to transfer useful abilities from one strain to another:

*Clostridium acetobutylicum* ATCC 824 has been equipped with genes from *C. thermocellum* for the formation of active mini-cellulosomes [151, 318]. Strains equipped with both a functional cellulosome and enzymes for the formation of butanol would allow solvent formation directly from lignocellulosic biomass and enable the use of such a host in a consolidated bioprocess. The establishment of a functional WLP in *C. acetobutylicum* ATCC 824 was investigated as well [33, 77]. Activity could be demonstrated for both the Eastern and Western branch of the WLP. There was, however, a lack of carbon flux from the WLP to acetyl-CoA that was hypothesized to be caused by a low level of the enzyme acetyl-CoA synthase [77]. Integrating the WLP into solventogenic clostridia would allow to recapture the H_2_ and CO_2_ released during metabolization of carbohydrates and to increase the overall carbon yield.

### Extended genome scale metabolic models

A recent development was the integration of GSM models of the acetogens *C. ljungdahlii* and *C. autoethanogenum* into spatiotemporal models of large-scale (30–125 m^3^) bubble column reactors for gas fermentation [39, 40, 167]. These models enable prediction of cellular performance considering spatially resolved gradients of solved substrate gases (H_2_, CO and CO_2_) in the reactor environment. An integrated GSM model was used to investigate targets for gene knockouts that improve cellular performance in industrial scale [40]. There also exist other models of large-scale bubble column fermentation where the biology was modeled with a fundamental set of reactions [269] or with a biothermodynamics approach [7]. Considering the industrial importance of bubble column reactors for gas fermentation [39, 275, 284], the rise of these models supports further scale-up and industrialization of gas fermentation.

Another exciting advancement is the development of a metabolism and macromolecular synthesis model (ME-model) including protein and RNA synthesis in a GSM model [177]. The obtained model is the first of its kind for gram-positive bacteria and shows an improved prediction of growth rate, acetate formation rate and production of reduced compounds such as ethanol and glycerol compared to the underlying GSM model. It also allows to model the influence of cofactor (Ni^+^) availability suggesting new applications like media optimization.

### Omics approaches

While GSM models can describe the general metabolic capabilities of an organism, gene expression varies depending on environmental conditions. The current metabolic phenotype can be accessed on proteome, transcriptome, and metabolome level with single- and multiomics approaches [285, 333]. Additionally, fluxomics approaches can use GSM models to calculate and estimate metabolite fluxes.

Single- and multiomics approaches have been applied to monitor the transition from solventogenesis to acidogenesis in solventogenic clostridia. The onset of solventogenesis and sporulation superimpose each other during batch cultivation. Continuous cultivation of *C. acetobutylicum* in a phosphate-limited chemostat allowed the culture to switch from acidogenesis to solventogenesis without triggering sporulation [97]. The possibility to investigate different metabolic states separately and reproducibly makes continuous cultivation a valuable tool for systems biology studies [97, 332, 333].

Naturally, studies aimed to understand phenomena that impair the industrial application and continuous cultivation of solventogenic and acetogenic clostridia. Regarding solventogenic clostridia, such phenomena include strain degeneration [131], solvent tolerance [110] and the response to inhibitors found in hydrolyzed lignocellulose [160, 176, 337]. A review about systems biology studies of *C. acetobutylicum* has been published recently and is highly recommended to the reader [333].

Several well-known acetogens such as *C. ljungdahlii, C. autoethanogenum, C. ragsdalei* and *C. coskatii* can produce acetate and ethanol simultaneously [19]. Ethanol is a desired product and formed acetate leaving the process is considered a “carbon loss” [301]. Several omics studies hence investigated the influence of the pH-value and substrate limitation [249] or the composition of the feed gas [106, 299, 300, 340] on ethanol formation. One interesting finding of proteome studies is that an increase in ethanol production seems not to be linked to key enzyme abundance in both *C. ljungdahlii* [249] and *C. autoethanogenum* [299], suggesting that regulation might be thermodynamically or on a posttranslational level rather than on a transcriptional level.

Systems biology approaches are also applied to investigate the function of key enzymes in metabolic pathways. Biochemical studies of relevant oxidoreductases in *C. autoethanogenum* cell extract in combination with transcriptome analysis allowed to determine the activity and the electron donor and acceptor specificity of key enzymes of the WLP and ethanol formation [198]. However, the activity of the methylenetetrahydrofolate reductase could only be demonstrated with the artificial electron acceptor benzyl viologen. Metabolic modelling employing a GSM model suggested that this enzyme is ferredoxin reducing, potentially filling this gap [300]. Recently, a GSM model of *C. drakei* coupled with transcriptome analysis and ^13^C metabolic tracing experiments was used to prove a functional cooperation of the glycine synthase-reductase pathway (GSRP) and the WLP [271]. The subsequent successful expression of the GSRP into *E. limosum* with a plasmid-based system underlines once again the modularity of metabolism.

### Strain engineering and design

The ever-improving understanding of the metabolism of acetogens and clostridia driven by systems biology promotes rational strain design. In addition to studies directly benefiting from mathematical and integrative system support [333], there are plenty of strain engineering studies with straight-forward approaches. Targeted properties include inhibitor tolerance for growth on complex feedstocks [160], increased productivity [124, 276, 335] product selectivity [158, 169, 310] and the expansion of the product spectrum [47, 108, 149]. Advances in metabolically engineered solventogenic clostridia and acetogens have been reviewed recently [41, 116, 149, 166, 208].

Important phenotypical properties for a robust solventogenic producer strain are abolished sporulation and increased solvent tolerance [166, 292]. These traits are especially important for continuous cultivation: sporulation associated with a halt of cell growth would lead to the cells washing out. The culture broth constantly contains increased solvent levels which cause cell stress. Even though clostridial butanol tolerance and its mechanisms are not completely understood to date [331], rational approaches have already been described to increase solvent tolerance [188, 323]. While rationally engineered strains showed a more rapid adaptation to butanol or performed better than the wild type when challenged with butanol, performance above the critical level of 2% (v/v) butanol were not tested [188] or could not be overcome [323]. Rational design of asporogenous *C. acetobutylicum* strains focused on inactivating the sporulation regulators *σ*^F^, *σ*^E^, *σ*^G^ and SpollE [292]. Both deletion of SpollE and *σ*^G^ resulted in asporogenous strains that formed solvents in an inoculum independent manner [22, 293]. However, inactivation of SpollE led to lower final solvent titers as compared to the wild type [22].

Random strain engineering strategies have been a valuable alternative to rational approaches. The generation of a strain library via random mutagenesis and subsequent screening for better producers proved useful to isolate an improved strain: *C. acetobutylicum* ATCC 55025 is asporogenous and produces high concentrations of butanol and total solvents [122]. This strain was further evolved to the strain JB-200. *C. acetobutylicum* JB-200 is asporogenous, butanol tolerant and hyper-producing [324], showing that these properties are compatible in clostridia. Comparative genomic analysis of the *C. acetobutylicum* strains ATCC 55,025, JB-200 and ATCC 824 identified the orphan histidine kinase cac3319 as a knockout target for increased butanol production and tolerance [324]. Butanol stress has also been a major subject of multiomics studies [110, 333]. The improving knowledge on butanol tolerance and asporogenous strains paves the way for future rational strain design.

### Genome engineering

Clostridia are challenging hosts for genome engineering. Common challenges are their low transformation and recombination efficiency [135, 149]. Clostridia lack non-homologous end-joining (NHEJ) and show a low activity of homology-directed repair (HDR) [135], both cellular repair mechanism for DNA double-strand breaks. The low activity of repair mechanisms can be used to screen for homologous recombination events with donor DNA. The genomic integration site can be targeted with high sequence specificity using a CRISPR/Cas system. Integration of the donor DNA removes the sequence that is targeted by CRISPR/Cas and protects the cell with the modified genome from the introduction of a lethal double-strand break [195]. To exploit HDR itself for the genomic integration of donor DNA, better understanding of homologous recombination mechanisms in clostridia and acetogens is needed [37]. Despite the challenges, genome engineering has been a focus of recent research and significant progress has been made. The latest published genome engineering tools for clostridia are summarized in Table 3.

**Table 3:** Tools for genome engineering of clostridia

Purpose	Tool	Description	Application	References
Genomic integration of whole pathways	Phage serine integrase system for dual integrase casette exchange (DICE)	Allows integrase-mediated site-specific integration into the genome without integration of unwanted DNA-like plasmid backbones	The whole butyric acid production pathway was integrated into the *C. ljungdahlii* genome	[112]
Genomic integration of whole pathways	Genomic integration system based on the Himar1 transposase	The Himar1 transposase is used to integrate the target DNA casette randomly at any AT-site in the genome	The acetone production pathway and an *ermC* selectable marker were integrated into the *C. ljungdahlii* genome	[220]
Deletion of single genes	CRISPR nickase based system for deletion	The truncated Cas9 protein (trCas9) lacking the RuvCl nucleolytic domain can be used for deletions even when expressed strong and constitutively	Two *ermB* genes and *pyrE* were deleted from the *Clostridioides difficile* genome	[118]
Deletion and integration of pathways	Targetron-recombinase system for large-scale genome engineering	Targetrons are used to position markerless *lox66* and *lox71* sites in the genome. Cre recombinase deletes the DNA in between the *lox66* and *lox71* site via homologous recombination	A 50-gene prophage island was deleted from the *C. phytofermentans* genome	[35]
Complementation after deletion	CRISPR/Cas9-based complementation strategy employing 24 nt bookmark sequences	A 24 nt bookmark sequence is introduced at the place of a gene that has been deleted. For future complementation studies, the 24 nt bookmark sequence is selected against to integrate the wildtype gene at its original location	The *pyrE* gene in *C. ljungdahlii* was replaced with 9 consecutive bookmark sequences. All 9 bookmark sequences allowed complementation with the *pyrE* wildtype gene	[264]
Editing of single nucleotides in genome	CRISPR-targeted base editing via deamination	A combination of nuclease deactivated Cas9 with activation-induced cytidine deaminase is applied for cytosine to thymine substitution without DNA cleavage	Premature stop codons were introduced into genes related to the formation of acetate (*pta*) and ethanol (adhE1, adhE2, aor1, aor2) in *C. ljungdahlii*	[320]
Editing of single nucleotides in genome	CRISPR nickase assisted base editing via deamination	A fusion of cytidine deaminase, CRISPR-Cas9^D10A^ nickase and uracil DNA glycosylase inhibitor (UGI) is used for base-pair substitutions of C∙G to A∙T	Mutations were introduced into the *pyrE, xylR, Spo0A* and *araR* gene of *C. beijerinckii*	[163]

Large-scale genome engineering tools such as the deletion of whole prophage islands or the integration of whole metabolic pathways have been developed for clostridial systems [35, 112, 220]. These tools may be used for new applications like the generation of a library of genome reduced strains and to improve the fast engineering of stable producer strains. CRISPR-targeted base editing tools allow genome engineering while avoiding the need for homologous recombination events, the introduction of donor DNA and DNA double-strand breaks [163, 320]. A useful application for base editing tools is the introduction of premature stop codons into genes to disrupt the gene function.

The number of available tools for genome engineering and metabolic engineering of clostridia increased significantly over the past decade. Further tools including plasmid systems for gene overexpression, dCas9 and RNA systems for gene down-regulation and gene deletion and insertion tools are reviewed elsewhere [37, 135, 166, 195]. An impressive testimony for the importance and applicability of genetic engineering of anaerobic microorganisms is the custom-made ‘Clostridia Biofoundry’ for fully automated, high throughput strain engineering used by the commercial syngas fermenting company LanzaTech [106].

## Alternative feedstocks

Solventogenic and acetogenic clostridia offer the possibility to use a broad substrate range for fermentation processes. The choice of feedstock has a big impact on the economic viability of the solvent production process and the price of the final product [68, 232]. Since the main solvents butanol and ethanol are bulk chemicals, the feedstock should be cheap and available in large quantities [21]. While basic research mainly relies on costly glucose [14, 81, 204, 222], glycerol and crude glycerol [9, 23, 86, 187], the historical ABE fermentation process mainly utilizes sugar- and starch-rich first-generation feedstocks such as sugarcane, molasses and maize [92, 103, 212]. Alternative feedstocks offering a high potential are food and agricultural waste [2, 75, 236, 237], lignocellulosic biomass [54, 117, 137, 203], and liquid waste streams, for instance of the pulp and paper industry [102] (see Table 4). Waste streams and lignocellulosic feedstocks are abundant, cheap and not in competition with food production [201]. The use of alternative feedstocks is more sustainable and offers a lower carbon footprint by saving the waste streams from incineration and thereby decreasing the greenhouse gas emissions [32, 95]. The European Commission estimated around 88 million tons of food waste produced in Europe which equals 3.3 Gt of CO_2_ per year [2, 25, 274]. Food waste is defined as a waste of restaurants, canteens, and the food processing industry [2, 85]. Food waste mainly contains sugar and starch but also a large portion of fibers [2, 85]. Agricultural residues and plant-based biomass are also called second-generation feedstocks as they mainly contain lignocellulose [209]. Lignocellulosic biomass is woody and fibrous material composed of a complex structure of cellulose, hemicellulose and lignin [32, 92, 117].

**Table 4:** Overview of industrially relevant alternative feedstocks for solventogenic and acetogenic clostridia

Carbon source	Feedstock	Pretreatment	Organism	References
**Lignocellulosic biomass**				
C6 and C5 sugars	Apple pomace ultra-filtration sludge	Dilute sulfuric acid pretreatment and detoxification method	*C. beijerinckii* NRRL B-466	[186]
	Barley straw	Acid hydrolysis and overliming	*C. beijerinckii* P260	[236]
	Cassava bagasse	Mechanically milling, enzymatic hydrolysis	*C. acetobutylicum* JB200	[182]
	Corn stover	Hot-water with wheat straw hydrolysate and overliming	*C. beijerinckii* P260	[237]
		Enzymatic hydrolysis	*C. saccharobutylicum* DSM 13864	[213]
		Dilute sulfuric acid pretreatment	*C. beijerinckii* BA101	[67]
	Domestic organic waste	Steam explosion and enzymatic hydrolysis	*C. beijerinckii B-592*, *C. acetobutylicum* DSM 1731	[42]
		Extruded, enzymatic hydrolysis	*C. acetobutylicum* ATCC 824	[180]
	Market refused vegetables	Shredded	*C. acetobutylicum* DSM 792	[280]
	Municipal solid waste	Dilute acid or hot water treatment and enzymatic hydrolysis	*C. acetobutylicum* NRRL B-591	[75]
	Pine and elm woods	Enzymatic hydrolysis and organosolv pretreatment	*C. acetobutylicum* NRRL B-591	[8]
	Pineapple peel	Grounded, dried, saccharification, detoxification method	*C. acetobutylicum* B 527	[144]
	Rice straw	Enzymatic hydrolysis, alkaline and concentrated phosphoric acid pretreatments	*C. acetobutylicum* NRRL B-591	[202]
	Starch industry wastewater	Dilute sulfuric acid pretreatment and detoxification method	*C. beijerinckii* NRRL B-466	[186]
	Suspended brewery liquid waste	Dilute sulfuric acid pretreatment and detoxification method	*C. beijerinckii* NRRL B-466	[186]
	Switchgrass	Dilute sulfuric acid pretreatment, enzymatic hydrolysis	*C. beijerinckii* P260	[237]
		Alkali-pretreatment	*C. saccharobutylicum* DSM 13864	[87]
	Wheat straw	Grounded, hot dilute sulfuric acid hydrolysis	*C. beijerinckii* P260	[235, 238, 239]
	Wood pulping hydrolysate	Detoxification: ion exchange resins, overliming and activated charcoal adsorption	*C. beijerinckii* CC101	[181]
**Starch waste streams**				
Starch	Food waste	Shredding	*Clostridium* sp. BOH3	[334]
		Blending and drying	*Clostridium* sp. strain HN4	[224]
		Pulverization	*C. beijerinckii* P260	[113]
	Sago	Enzymatic hydrolysis	*C. saccharobutylicum* DSM 13864	[171]
	Starch-based waste packing peanuts	–	*C. beijerinckii* BA101	[129]
	Potato waste starch	–	*C. acetobutylicum* NRRL B-591	[145]
	Defibered-sweet-potato slurry	–	*C. acetobutylicum* P262	[12]
	Inedible dough	–	*C. beijerinckii* NCIMB 8053	[297]
**Sugar waste streams**				
Sucrose, fructose, raffinose, stachyose, verbascose	Soy molasses	–	*C. beijerinckii* BA101	[232]
Glucose, mannose	Konjac waste	Enzymatic hydrolysis, simultaneous saccharification and fermentation	*C. acetobutylicum* ATCC 824	[265]
Cellobiose, glucose	Waste cotton fibers	Phosphoric acid-acetone process and enzymatic hydrolysis	*C. acetobutylicum* NRRL B-591	[261]
Lactose	Milk dust powder	–	*C. acetobutylicum* ATCC 824, *C. beijerinckii* NCIMB 8052	[296]
	Cheese whey	–	*C. acetobutylicum* P262	[62, 63]
**Gaseous feedstocks**				
CO:CO_2_:H_2_:N_2_ (16.5:15.5:5:56)	Gasified Switchgrass	Ash removal by cyclone, scrubbers with 90% water, 10% acetone	*C. carboxidivorans*	[6]
CO:CO_2_:H_2_:N_2_ (42:20:2:36)	Steel mill waste gas	–	*C. autoethanogenum*	[198]
CO:CO_2_:H_2_:N_2_ (44:22:2:32)		–	*C. autoethanogenum*, *C. ljungdahlii*, *C. ragsdalei*	[148]
CO:H_2_:CO_2_ (40:30:30)	Syngas	–	*Clostridium ragsdalei* PTA-7826	[277]
CO:CO_2_:H_2_ (10:60:30)		Electrolysis of CO_2_ and H_2_O to form CO and H_2_	*C. autoethanogenum*, *C. kluyveri*	[104]

While sugar substrates can be directly used in fermentation processes, feedstocks containing starch are primarily saccharified to glucose by glucoamylase [75, 289]. However, there are clostridia which can directly utilize starch, such as *C. acetobutylicum* NRRL B-591 and *Clostridium beijerinckii* BA101 [68, 71, 145, 184]. Therefore, food wastes are easily accessible and do not require expensive pretreatment [2, 113]. Conversely, feedstocks with a high lignocellulosic fraction such as wheat straw, corn stover, rice straw and cassava bagasse (see Table 4) require a pretreatment to release the sugars for conversion [32, 203]. Likewise, hydrolysis and/or saccharification can be integrated into the fermentation [32, 137]. For detailed information about pretreatment and integrated methods, the reader is referred to recent reviews on this topic [21, 32, 92, 117]. According to Ibrahim et al. [117], pretreatment and integrated methods increase capital and operational costs as well as time and energy requirements. Cao and Sheng [32] additionally underlined the negative effect of degradation and loss of carbohydrates. Sugar degradation not only decreases the proportion of convertible sugars but also leads to the formation of toxic compounds (e.g. furfural and 5-hydroxymethylfurfural), which may inhibit cell growth and lower the productivity of the process [32]. To decrease toxicity, hydrolysates can be treated to remove inhibitors prior to fermentation [32]. Liquid waste streams such as soy molasses [232], cheese whey [242] and Kraft paper mill sludge [102] are advantageous as they are already rich in free sugars and do not require hydrolysis. However, some liquid waste streams like paper mill sludge require detoxification to reduce growth-inhibiting components [95].

Saccharified lignocellulose and waste streams of the pulp and paper industry, contain a sugar mixture of hexoses (e.g. glucose, galactose, fructose) and pentoses (e.g. xylose, arabinose) [289]. For high productivity and an economic-efficient production process total sugar utilization is essential [168]. Unlike most natural yeast strains, solventogenic clostridia are particularly well suited to ferment pentose sugars like xylose [338, 343]. Despite the ability to convert a broad spectrum of sugars, the well-known problem of carbon catabolite repression (CCR) in sugar mixtures is a remaining issue. Therefore, recent studies have focused on the efficient conversion of sugar mixtures [289, 292]. Current research wants to go further by focusing on strains naturally capable to degrade cellulose and the genetic modification of the metabolic pathways. The major goal is the direct conversion of the complex structured lignocellulosic biomass to avoid expensive pretreatment steps [123]. For more information about genetic modification of metabolism, the reader is referred to the section “Systems biology and genetic engineering” and Jang et al. [123].

Since acetogens came to the center of attention, there are far more possibilities using alternative feedstocks: acetogenic clostridia can not only grow heterotrophically on a range of carbon sources but also autotrophically on gaseous substrates [192]. Gas mixtures of CO, H_2_ and CO_2_ are suitable substrates for gas fermentation of acetogens. These gas mixtures referred to as synthesis gas or syngas can be sustainably produced by the gasification of lignocellulosic biomass and municipal solid waste (MSW) [170]. Gasification yields accessible carbon even from the complex lignin fraction that accounts for up to 40% of the plant biomass [278]. Other sources of syngas include industrial waste streams such as exhaust gas of the steel and oil industry [270] and even gas mixtures obtained electrochemically from CO_2_ and H_2_O [104, 275].

While no fixed ratio of H_2_/CO is needed for syngas fermentation [11], the overall gas composition does influence the bioprocess. A higher ratio of H_2_ to CO may reduce the loss of carbon as CO_2_ and influence the product spectrum [170, 299]. The composition of the gas mixture depends on its origin. Syngas obtained from biomass gasification as well as furnace gas from steel mills may contain several detrimental impurities including ammonia (NH_3_), nitrogen oxide (NOx) and other enzyme inhibitory compounds such as acetylene (C_2_H_2_), ethylene (C_2_H_4_), ethane (C_2_H_6_) and oxygen (O_2_) [107]. The presence of the inhibitor hydrogen cyanide (HCN) in the feed gas even forced a temporal shutdown of a semi-commercial plant for ethanol production from gasified biomass and MSW [303]. Some impurities may also influence process parameters such as the pH-value, the osmolarity or the oxidation-reduction potential (ORP) [322]. Cleanup methods for removal of different impurities are available but costly and should be reduced to the minimum [48].

CO and H_2_ are poorly soluble in water (83 and 71% of the solubility of oxygen at 37 °C, respectively [221]) and must be continuously transferred from the gaseous to the liquid phase during gas fermentations. A high mass transfer of gases into the liquid is desired to enable high production rates and near-complete conversion of the feed gas. Unconverted gas leaving the bioreactor means both loss of valuable substrate and emission of greenhouse gas (GHG) [303].

ANP mixotrophy is a common feature of acetogens allowing them to utilize gaseous substrates and organic carbon sources simultaneously [134]. An increase in carbon yield from carbohydrates through ANP mixotrophy has been demonstrated for several acetogens [134] [27, 192] and a patent for the mixotrophic production of butanol, butyrate, isopropanol, acetone and ethanol has been issued [294]. A next step towards industrial application would be to demonstrate the benefits of mixotrophy during growth on complex feedstocks like hydrolyzed lignocellulose.

In conclusion, the use of alternative feedstocks with solventogenic clostridia has been much better researched compared to acetogenic clostridia. With the ability to co-utilize gaseous and organic substrates, however, acetogens seem an attractive option to develop carbon efficient bioprocesses with superior product yields from cheap carbon and energy sources. After focusing on the organism in combination with a cheap and sustainable feedstock for high efficiency of solvent production, the next step is the technical side of process optimization.

## Continuous fermentation methods

In this section, we pay special attention to the different operation strategies for continuous fermentations, their properties and potential as a powerful tool to develop solvent production towards industrial implementation. For the design of a new economical process, the choice of the reactor type and the operation strategy are the two major criteria, mostly affecting the formation and activity of biocatalyst, conversion rate, volumetric productivity and downstream processing [179, 275, 314].

### Batch and fed-batch

The batch process is easy to operate and requires minimum control. For that reason, it was conventionally used for the first laboratory studies and industrial ABE processes in Europe [21, 133, 179, 343]. Compared to fed-batch and continuous mode, batch mode reached the highest solvent yield for ABE fermentation [165]. However, changing conditions in batch over time (e.g. product concentration) can lead to an uncontrolled switch between the acidogenic and solventogenic phase, inhibited growth or cell death [105, 133, 179, 200]. Major drawbacks for industrial use of the batch mode are downtime periods for reactor preparation and prolonged lag phases leading to an overall low productivity [43, 161, 179].

When referring to batch mode during gas fermentations, the liquid volume remains unchanged, while the gaseous substrate is typically supplied either at the beginning (batch mode) or as continuous flow (fed-batch) [98, 276, 340]. While bioreactors offer control and monitoring possibilities [140], serum bottles represent the only “real” batch cultivations in gas fermentation, delimiting gas exchange and stripping of (intermediate) products.

Feeding strategies in fed-batch mode give the possibility to maintain a certain growth rate and low substrate concentration which offers the use of substrates toxic to the cells in large amounts and to obtain higher biomass and product concentrations than in batch cultivations [70, 92, 179, 226, 227]. Accumulation of products (like butanol) to toxic levels in the fed-batch process can inhibit the growth and product formation. A significantly improved solvent productivity was achieved by the integration of product recovery [70].

Nevertheless, the downtime in a fed-batch is comparable to a batch process and likewise there is no continuous substrate conversion and product formation. Multiple studies have investigated the use of fed-batch in comparison to continuous processes [124, 174, 182, 239, 288, 340]. Li et al. [165] tested batch, fed-batch and continuous process modes for ABE fermentation and recommended continuous fermentations to obtain bioprocesses with superior productivities.

### Continuous processes

In contrast to batch and fed-batch cultivations, continuous cultivations are more demanding in terms of process control but offer significantly higher productivity and advanced capabilities for process design. Increased efficiency in industrial scale is offered, due to minimal initial lag phase, possible continuous feeding of permanently accumulating waste streams, steady downstream processing and thereby reduced downtime [16, 72, 92, 95, 107, 164, 179, 343]. Compared to short batch cultivations, continuous processes require increased attention to maintain strictly anaerobic conditions and to avoid microbial contaminations [161, 247, 343]. Table 5 gives a quick overview of the advantages and disadvantages of the continuous fermentation methods and operational strategies for solvent production described in the following sections.

**Table 5:** Comparison of the most advantageous continuous fermentation methods and configurations for solvent production with solventogenic and acetogenic clostridia

Method/configuration	Advantages	Disadvantages	References
One-stage chemostat	Maintain growth rate at defined value Supports growth-related products Stable gas fermentation with acetogens	Maximum growth rate limited by dilution rate Low biomass during solvent formation Strain degeneration and difficulty to reach steady state conditions with solventogenic clostridia	[18, 193, 319]
Multi-stage systems	Tool to stabilize biphasic fermentations Variation of temperature, pH or nutrient supply between the stages	Higher costs for multiple reactors Complex control	[18, 205, 247, 275, 279]
Cell retention	Uncouples dilution rate of specific growth rate High volumetric productivity Full control of biocatalyst concentration Increased conversion rates (Toxic) solvents can easily be recovered of cell-free permeate Circulation of effluent possible Biomass reuse lowers propagation costs	Difficult long-term operation Costly membrane Membrane fouling At high-level biomass concentration operational problems (high viscosity, heavy gas formation and foaming) Higher contamination risk of external separation Nonselective retention (dead, non-viable cells, and substrate particles) Requirement of cell viability monitoring	[21, 62, 161, 164, 185, 204, 222, 287, 308, 314]
Cell immobilization and biofilm reactors	Prevents washout of cells Allows higher dilution rates Increases reaction rates and productivity Enhanced genetic stability Improved inhibitor resistance of cells Protects cells against shear forces	Uncontrolled cell growth can lead to blocking or Membrane fouling Maintenance of cell viability and physiology Diffusion limitation of mass-transfer Varying microenvironment Leaking of cells of support Inactive or dead biomass Reduced productivity during longer-term operation Challenging scale-up	[11, 138, 150, 168, 179, 205, 225, 231, 275, 284, 314, 341]
Integrated product recovery	Energy-efficient at low solvent concentrations Integration of downstream step for solvent recovery into cultivation Operable in continuous mode Lowers the concentration of toxic products in the broth Decreased product inhibition Improved substrate conversion rates, solvent yields, and productivities In situ product recovery: culture broth does not leave the reactor	In-line method in separate recovery loop affecting the cells In situ product recovery: limited optimization opportunities Disadvantages dependent on product recovery method	[21, 58, 82, 161, 164, 306, 325]

### One-stage chemostats

Stable continuous fermentation in chemostat was successfully maintained in several publications [13, 23, 43, 127, 307]. A commonly referred strain in stable chemostat runs is *Clostridium acetobutylicum* ATCC 824 [9, 90, 126, 127, 272]. For instance, more than 70 days of stable chemostat cultivation of *C. acetobutylicum* ATCC 824 was achieved at pH 6 and a dilution rate of 0.05 h^−1^ with a substrate-mixture of glucose and low-grade glycerol [9]. Butanol was the major solvent, produced with a yield of 0.34 mol mol^−1^ and a productivity of 0.42 g L^−1^ h^−1^, one of the highest reported productivity values for chemostat cultivations with *C. acetobutylicum* [9].

Basic lab-scale approaches for gas fermentation were mainly applied in continuous cultivation [84, 106, 146, 199, 275, 299, 300]. The continuous gas fermentation leads to a steady value of dissolved gases in the liquid medium which allows a precise calculation of the substrate consumption rate by monitoring the off-gas composition. The continuous stirred-tank reactor (CSTR) offers extensive mixing capabilities by the steady distribution of gaseous and liquid substrates [28, 107, 275]. The resulting high mass transfer rate is the reason why CSTRs are the first choice for gas fermentation investigations [4, 11, 106, 107, 170, 199, 217, 275, 298, 299]. For industrial-scale gas fermentations, the energy demand for sufficient mixing is significantly increased in CSTRs. As an alternative bubble columns, gas lift and loop reactors showed to be simple and cost-efficient, with the possibility for an energy-efficient scale-up [96, 275, 284]. However, for solvent production from organic substrates with suspended cells, the CSTR is still the dominating reactor type in industrial scale.

Continuous bioprocessing with solventogenic clostridia is challenging due to strain degeneration and because steady-state conditions can be difficult to establish [16, 18, 131, 141, 319, 343]. In the past, the degeneration of different *Clostridium acetobutylicum* strains (ATCC 824, DSM I73, NCIB 8052 and P262) in chemostat cultivations was investigated in multiple studies [9, 272, 319]. It has been shown that degenerated and solventogenic clostridia are transiently in co-culture but with increased cultivation time the fast-growing degenerated cells outgrow the slow-growing solvent-producing cells [45, 70, 95, 319]. One possible explanation is the strong selection pressure acting on the cells in a long-term cultivation. The increased number of generations, compared to a batch process, is not only detrimental for the genetic stability of genetically engineered organisms but also increases the chance for natural and induced mutations [179, 193].

One-stage chemostats for ABE fermentation often failed to reach steady-state conditions and are marked by the oscillation of biomass, product, and substrate concentration [18, 86, 204]. So far, the influence of culture pH, extracellular addition of butyric acid or acetic acid as co-substrate and phosphate (P) or nitrogen (N) limitations on culture stability has been investigated [13, 46, 105, 130, 200, 343]. Although nutrient limitations can efficiently stabilize cultures, this stability can only be achieved at the expense of incomplete carbon substrate utilization. In contrast to solventogenic clostridia, acetogenic microorganisms easily reached the steady-state in one-stage chemostats and strain degeneration has never been reported. A conclusion on ABE fermentation may be drawn by comparing solventogenic clostridia and acetogens on the systems biological level.

During chemostat cultivation, the close link of the volumetric productivity to the liquid dilution rate and thus, the specific growth rate, offers higher process control. However, the maximum growth rate of the cells limits the dilution rate. While a chemostat process is advantageous for growth-related products, growth inhibition by toxic products and low growth rates during solventogenesis result in a limitation of the dilution rate [26, 161, 165, 166, 179]. Low biomass concentrations were also reported for gas fermentations with acetogens [37]. Low cell concentrations in combination with low dilution rates eventually limit the volumetric solvent productivity in the chemostat. Optimization of processes with solventogenic and acetogenic clostridia, therefore, requires additional modifications of the basic one-stage chemostat, described in the following sections.

### Multi-stage systems

**Fig. 2:**
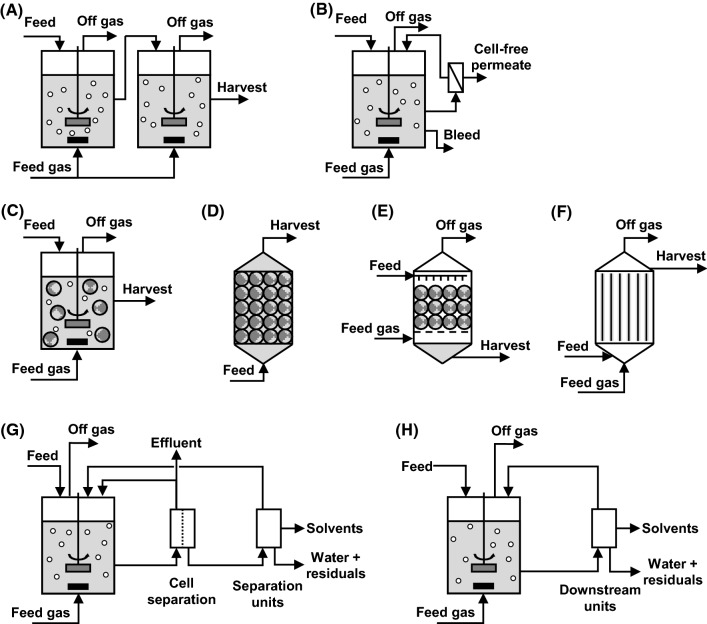
Overview on the most advantageous fermentation methods and configurations for continuous solvent production with solventogenic and acetogenic clostridia. **a** Multi-stage process with two chemostat stages; high cell density cultivation in a **b** continuous cell retention system and with **c**–**f** immobilized systems and biofilm reactors: **c** chemostat with free-flowing immobilized cell particles, **d** packed-bed reactor (PBR), **e** trickle bed reactor (TBR), **f** hollow fiber membrane reactor (HFMBR). TBR (**e**) and HFMBR (**f**) are mainly used for gas fermentation. Integrated product recovery methods: **g** in-line recovery and **h** in situ recovery. (Modified from [82, 267, 275, 314, 341]

A technical solution for stabilization of the continuous production are multi-stage systems where multiple reactors connected serially form a “reactor cascade” (see Fig. 2a). For example, the process can be split into a nutrient-limited phase (e.g. phosphor or nitrogen) and a solvent forming phase by variation of temperature, pH or nutrient supply between the stages (see Table 6) [18, 88, 154, 205, 279]. Two-staged reactor systems were proven to enhance the stability of the cell physiology and product formation of solventogenic fermentations, either with heterogenic or gaseous substrates [18, 205, 247, 275]. The use of continuous two-stage chemostats for solventogenic clostridia was first discussed by Bahl et al. [14] and has subsequently been investigated as a tool to stabilize biphasic fermentations [18, 91, 154, 205].

**Table 6:** Application of the multi-stage process for continuous solvent production with solventogenic and acetogenic clostridia

Strain	Continuous system	1st stage	2nd stage	Substrate	Product	Titer, productivity, yield	References
*C. acetobutylicum* DSM 1731	Two-stage chemostat	D 0.125 h^−1^, P-limited	D 0.04 h^−1^, P-limited	Glucose	Butanol	130 mM	[14]
					Acetone	74 mM	
*C. acetobutylicum* IFP 918	Two-stage chemostat	D 0.16 h^−1^, pH 6.1, T 36 °C	D 0.045 h^−1^, pH 5.5, T 33 °C, N-limited	Glucose	ABE solvents	0.28 g g^−1^	[18]
*C. acetobutylicum*	Two-stage fermentation	D 0.08 h^−1^, pH 4.5	D 0.04 h^−1^, pH 4.5–5.0	Glucose	ABE solvents	21 g L^−1^, 0.36 g g^−1^	[91]
*C. acetobutylicum* ATCC 824	Two-stage chemostat	D 0.075 h^−1^, pH 6.0, T 37 °C, N-limited, acid formation	D 0.06 h^−1^, pH 4.5, T 33 °C, solvent production	Glucose	ABE solvents	9.11 g L^−^1, 0.6 g L^−1^ h^−1^	[154]
					Butanol	5.93 g L^−1^, 0.4 g L^−1^ h^−1^	
*C. acetobutylicum* ATCC 824	Two-stage turbidostat/ chemostat	pH 4.62, acid formation	pH 4.37, solvent production and in situ product recovery	Glucose	ABE solvents	18.0 g L^−1^, 0.13 g L^−1^ h^−1^, 0.30 g g^−1^	[305]
*C. acetobutylicum* B 5313	Two-stage chemostat and cell immob., in situ product recovery	D 0.6 h^−^1, 37 °C	D 0.3 h^−1^, 37 °C	Glucose	ABE solvents	25.32 g L^−1^, 2.5 g L^−1^ h^−1^, 0.35 g g^−1^	[17]
*C. beijerinckii* NRRL B592	Two-stage cascade and cell immobilization	D 0.5–0.6 h^−1^, pH 4.6–4.7, T 36 °C	D 0.15–0.20 h^−1^, pH 4.7–4.8, T 36 °C	Glucose	ABE solvents	9.27 g L^−1^, 1.24 g L^−1^ h^−1^	[88]
					Butanol	5.57 g L^−1^	
*C. beijerinckii* NRRL B592	Two-stage cascade	Turbidostat, D 0.12 h^−1^, pH 4.7, 34 °C, acid formation	Chemostat, D 0.022 h^−1^, pH 4.7, 34 °C, solvent production	Glucose	ABE solvents	15 g L^−1^, 0.27 g L^−1^ h^−1^	[205]
					Butanol	9.1 g L^−1^	
*C. beijerinckii* DSM 6423	Two-stage chemostat	D 112 h^−1^, pH 4.8	D 0.054 h^−1^, pH 5.1	Glucose	ABE solvents	10.56 g L^−1^, 0.39 g L^−1^ h^−1^	[279]
*C. ragsdalei* ATCC PTA-7826	Two-stage cascade with cell recycle and nutrient limitation	D 0.28 mL min^−1^, acid formation	D 0.28 mL min^−1^, ethanol production	Syngas: 30% H_2_, 30% CO_2_, 40% CO	Ethanol	(14.74 g g^−1^ cells)	[153]
*C. ljungdahlii* ERI-2 (ATCC 55380)	Two-stage cascade	CSTR, pH 5.5–5.7, growth stage	Bubble column, pH 4.4–4.8, with cell and gas recycle ethanol production	Syngas: 60% CO, 35% H_2_, 5% CO_2_	Ethanol	450 mM, 0.37 g L^−1^ h^−1^	[247]
*C. ljungdahlii* PETC	Two-stage chemostat	CSTR D 0.96 day^−1^, pH 5.5	Bubble column with cell recycle, D 0.48 day^−1^, pH 4.5	Syngas: 60% CO, 35% H_2_, 5% CO_2_	Ethanol	19 g L^−1^, 0.30 g L^−1^ h^−1^	[191]
*C. ljungdahlii* PETC	Two-stage chemostat	Acid formation	Ethanol production	Syngas: 60% CO, 35% H_2_, 5% CO_2_	Ethanol	188.2 mM	[249]

The table gives an overview of the settings of the most investigated two-stage process, in detail: dilution rate (D), pH, temperature (T), nutrient limitation (P: phosphate, N: nitrogen) and purpose of the respective stage

In 1998, ButylFuel LLC (Columbus, USA) patented a two-stage fermentation process separating acidogenesis and solventogenesis in two distinct process steps. In the first stage, *C. tyrobutyricum* converts glucose to butyric acid which is transferred to the second stage and converted to butanol by *C. acetobutylicum* [243].

Multi-staged processes are the method of choice in semi-continuous industrial ABE fermentation in Russia and China [95]. While ABE processes in Europe were merely focused on batch cultivation in the past, China and Russia continually focused on continuous bioprocessing to produce acetone, butanol, and ethanol [211, 343].

Recently, Richter et al. [247], and Martin et al. [191] applied a two-stage cultivation system for syngas fermentation, separating the process in a growth stage and an ethanol producing stage (see Table 6). In 2016, a continuous multi-stage cultivation in circulated loop reactors for gas fermentation was patented by LanzaTech [295], emphasizing the feasibility and suitability of multi-stage processes for industrial use.

Recent investigations of solventogenic clostridia demonstrate the continuous two-stage cultivations with integrated product recovery in the second stage [17, 304, 305]. As shown in Table 6, there are several possibilities to combine the reactor cascade with other technologies, such as cell recycling [5, 247] and cell immobilization [17, 88]. Cell recycling and immobilization can consolidate the idea of a growth and solvent forming process phase, as described for gas fermentation [247].

### High cell density cultivation

To solve the problem of insufficient biomass in continuous cultivations, growth needs to be uncoupled from the liquid feed flow rate. Uncoupling can be done by regulating the cell concentration in a continuous culture equipped with a cell retention technique or by immobilization of the cells [86, 114, 210, 222, 338]. The topics of cell retention and immobilization are described in the following sections.

#### Cell retention

The introduction of a cell retention or cell recycling unit uncouples the dilution rate from the specific growth rate and therefore allows to accumulate higher biocatalyst concentrations [161, 185, 314]. That way, a ‘retentostat’ offers the possibility of a fully controlled high cell density fermentation by increasing conversion rates for complete substrate uptake and efficient conversion into the target product. Cell retention has been reported to be advantageous for solventogenic clostridia and enables high volumetric productivity during gas fermentation with acetogens [37, 108, 134, 247, 248].

Cell retention with submerged cells can industrially be achieved by centrifugation and filtration, while membrane filtration is primarily used in lab-scale experiments [179, 314]. Using membrane filtration, biomass is increased by holding back the cells by a hollow-fiber membrane module (see Fig. 2b) [62, 222, 308]. The growth rate in the retentostat can be controlled by the value of bleed flow [185]. Of the obtained cell-free permeate, toxic solvents can easily be recovered, while the leftover substrate can be returned to the reactor for an increased conversion [62].

Systematic reuse of biomass can lower the costs of cell propagation [314]. On the other hand, the process may be more complex and difficult to operate in the long-term [62]. The requirement of a membrane for the cross-flow filtration increases the process costs and implies the risk of membrane fouling over time [21, 62, 287]. Cell recycling can be combined with different reactor types such as bubble columns and process modifications such as cell immobilization, latter reduces problems with membrane fouling [21, 173]. The use of an external separation method constitutes a higher risk for contamination compared to a conventional chemostat process. Rapid pumping of the cell broth through the separation device can cause cellular shear stress [204]. The use of a separation unit in industrial gas fermentations can lead to a deficit in gas supply due to longer residence times.

Cell retention has already been demonstrated in the past to increase the productivity in ABE fermentation of glucose by *Clostridium acetobutylicum* [5, 81, 204, 222, 257]. When research in ABE got back into the focus between 2005–2010, the topic of cell retention was rediscovered. Tashiro et al. [287] maintained a high cell density culture of *C. saccharoperbutylacetonicum* N1-4 in a membrane cell-recycling reactor, feeding glucose and showed an ABE productivity of 7.55 g L^−1^ h^−1^ and concentration of 8.58 g L^−1^ for more than 200 h without cell degeneration. More than 710 h of stable cell recycling application and conversion of glycerol to a high butanol productivity was shown with the hyper producing *Clostridium pasteurianum* MBEL_GLY2 [187]. Jang et al. [125] and Nguyen et al. [210] showed some of the highest achieved butanol productivities with 21.1 and 14 g L^−1^ h^−^1, respectively (see Table 7). Successful implementation of cell retention for the utilization of C5 sugars like xylose was shown by Zheng et al. [338] and Survase et al. [283].

**Table 7:** Application of cell retention for continuous solvent production with solventogenic and acetogenic clostridia

Strain	Continuous system	Dilution rate	Bleed rate	CDW	Substrate	Product	Titer/productivity /yield	References
*C. acetobutylicum* ATCC 824	Membrane cell-recycle reactor	0.5 h^−1^	0.025 h^−1^	20 g L^−1^	Glucose	ABE solvents	13 g L^−1^, 6.5 g L^−1^ h^−1^	[222]
*C. acetobutylicum* ATCC 824	Cell-recycle reactor	0.35 h^−1^	Total cell retention	125 g L^−1^	Glucose	ABE solvents	4.5 g L^−1^ h^−1^, 0.31 g g^−1^	[81]
						Butanol	3.7 g L^−1^ h^−1^	
*C. acetobutylicum* ATCC 824	Spin filter perfusion bioreactor	0.089 h^−1^	Total cell retention	49 g L^−l^	Glucose	ABE solvents	1.14 g L^−1^ h^−1^	[204]
*C. acetobutylicum *AvapClo™ (ATCC 824 with ADH gene)	Membrane cell-recycle reactor with product recovery of the permeate and effluent recirculation	0.77 h^–1^	Bleed for constant CDW	Max 50 g L^−1^	C6 lignocellulosic sugars from pine wood	Butanol, acetone, isopropanol and ethanol mix	10 g L^−1^ h^−^1, 0.33 g g^−1^ sugars	[283]
*C. acetobutylicum* BKM19	Membrane cell-recycle reactor	0.86 h^−1^	0.04 h^−1^	(OD_600nm_ 335)	Glucose	ABE solvents	23.5 g L^−1^, 21.1 g L^−1^ h^−1^, 0.34 g g^−1^	[125]
						Butanol	11.9 g L^−1^, 10.7 g L^−1^ h^−1^, 0.17 g g^−1^	
*C. acetobutylicum* CAB1060	Cell-recycle reactor with in situ extraction and P-limitation	0.076 h^–1^	0.05 h^−1^	28–30 g L^−1^	Glucose	Butanol	550 g L^−1^, 14 g L^−1^ h^−1^, 0.35 g g^−1^	[210]
*C. acetobutylicum* P262	Membrane cell-recycle reactor	0.41 h^−1^	0.02 h^−1^	20 g L^−1^	Cheese whey permeate	ABE solvents	0.31 g g^−1^	[62]
*C. acetobutylicum*	Cell-recycle reactor with P-limitation	0.40 h^−1^		13.1 g L^−1^	Glucose	Butanol	4.1 g L^−1^ h^−1^	[257]
*C. saccharoperbutyl-acetonicum* N1-4	Membrane cell-recycle reactor	0.71 h^−1^	0.16 h^−1^	16.3 g L^−1^	Glucose	ABE solvents	8.66 g L^−^1, 7.54 g L^−1^ h^−1^	[287]
*C. saccharoperbutyl-acetonicum* N1-4	Membrane cell-recycle reactor	0.78 h^−1^		17.4 g L^−1^	Xylose	Butanol	4.26 g L^−^1, 3.32 g L^−1^ h^−1^	[338]
*C. autoethanogenum* DSM10061	Membrane cell-recycle reactor	4.9 day^−1^	0.5 day^−1^	1.83 g L^−1^	65% H2, 23% CO_2_, 9%N_2_	Ethanol	6.3 g L^−1^, 0.14 M	[198]
*C. ljungdahlii ∆*SADH (pTCtA)	Cell-recycle reactor	3–6 mL min^−1^	Bleed for constant CDW	10–18 g L^−1^	Fructose	Acetone	10.8 g L^−1^	[134]
Co-culture: *C. ljungdahlii* PETC and *C. kluyveri* DSM555	Membrane cell-recycle reactor	40–80 mL h^−1^	10–20 mL h^−1^	(OD_600nm_ 5–10)	Syngas: 60% CO, 35%H_2_, 5% CO_2_	Ethanol	65.5 mmol CL^−1^ day^−1^	[248]
						Butanol	39.2 mmol CL^−1^ day^−1^	
						Hexanol	31.7 mmol CL^−1^ day^−1^	
*C. pasteurianum* MBEL_GLY2	Cell-recycle reactor	0.90 h^−1^	Total cell retention	(OD_600nm_ 407.6)	Glycerol	Total solvents	9.2 g L^−1^, 8.3 g L^−1^ h^−1^	[187]
						Butanol	8.6 g L^−1^, 7.8 g L^−1^ h^−1^	

The table focuses on cell recycling systems and shows the essential settings of dilution rate and bleed rate, deployed to achieve high cell dry weight (CDW)

The next step in research with solventogenic clostridia will be the optimized bioprocessing of alternative feedstocks. Liquid waste streams and pretreated substrates like lignocellulose hydrolysates comprise of a mixture of sugars but furthermore can contain a high solid particle concentration and inhibiting substances, leading to decreased cell growth. While cell retention is essential for efficient conversion of this kind of substrates, there may be an upcoming problem: the retention system is not selective for active biomass. Therefore, inactive cells and even substrate particles accumulate equally in the reactor. Consequently, an increase in biomass concentration does not necessarily lead to a proportional increase in productivity [308]. A major approach is the viability monitoring of the cell population and differentiation between cells and background particles via rapid at-line tools such as flow cytometry [291, 308].

Multiple studies showed the implementation of cell retention in (syn)gas fermentation with acetogens [36, 108, 178, 247, 248] (see Table 7). Additionally, Jones et al. [134] successfully showed mixotrophic growth of an engineered *Clostridium ljungdahlii* strain on syngas and fructose in a cell retention system. Regarding industrial production at scale, there are several patents for gas fermentation equipped with cell retention [84, 260].

#### Cell immobilization

Another option for continuous high cell density cultivation is the use of cell immobilization and biofilm reactors to prevent cell washout [168, 275, 314]. To that end, immobilization allows operation at higher dilution rates which in turn increases reaction rates and productivities [179, 225]. Advantages of immobilization include enhanced genetic stability, improved resistance of cells to inhibitory substrates or products and protection against shear forces [150, 341].

Immobilization is commonly achieved by entrapment of cells or by binding of cells to a carrier [341]. Cells can be entrapped inside a semipermeable membrane or encapsulated inside a polymeric matrix, for example inside beads of alginate or polyacrylamide (see Fig. 2c) [12, 145, 168, 179, 341]. Binding of the cells to the surface of a solid material is implemented by physical adsorption, ionic bonds, covalent bonds, or a mixture [55, 225, 341]. Entrapment and covalent bond formation require expensive and cell propagation limiting chemicals [179, 225]. In contrast, adsorption on a carrier is more natural, forms stronger bonds and can easily be performed in place [225]. A trend in research of immobilized solvent production is the use of cheap, renewable materials as adsorption carrier such as wood pulp [15], sugarcane bagasse [17], coconut fiber [281], corn stover [86] or clay bricks [240]. During adsorption, cell growth occurs in biofilms [179, 225, 231, 234].

Typical bioreactors for the bioprocessing with floating immobilized cells are CSTR (see Fig. 2c), fluidized bed bioreactors and air-lift reactors [341]. Packed bed reactors (PBR) differ from bioreactors with fully suspended culture as they are tightly packed with a carrier material to support biofilm formation (see Fig. 2d) [16, 225]. For gas fermentation, two special types of immobilized reactors have recently been described: the trickle bed reactor (TBR, see Fig. 2e) and the hollow fiber membrane reactor (HFMBR, see Fig. 2f) [11, 275, 295]. TBR are similar packed as PBR but the bed is sprinkled with liquid nutrient medium from above and flushed with the substrate gas from below to obtain high gas–liquid transport rates with low energy consumption [275]. A microporous membrane is used in a HFMBR for gas distribution and at the same time as carrier surface, providing cell growth at the gas–liquid interface with high mass transfer rate [267, 275]. Uncontrolled cell growth can lead to blocking of the PBR and TBR column, which was reported as a major problem in the first scale-up of the PBR process with solventogenic clostridia [225, 231, 275]. Moreover, membrane fouling of the cost-intensive membranes of HFMBR is a problem which causes the loss of membrane functionality [138, 275]. Immobilization leads to varying microenvironmental conditions and diffusion limitation of substrates and products, either by the thickness of the biofilm, pore size or surface area of the material [138, 179, 341]. The impeded mass transfer leads to inactive or dead biomass and a reduction of the volumetric productivity during Longer operation periods [204].

Several investigations with different cell immobilization techniques were performed over the years with solventogenic clostridia and in recent years with acetogenic gas fermentation (listed in Table 8). Gallazzi et al. [86] used a continuous immobilized packed-bed reactor filled with corn stover pieces for biofilm adsorption of *C. pasteurianum* DSM 525. During steady-state with 0.44 h^−1^ dilution rate, they reached a butanol titer of 10.4 g^−1^ L^−1^, productivity of 4.2 g^−1^ L^−1^ h^−1^ and 33% butanol to liquid by-products ratio. For syngas fermentation (38% CO, 28.5% CO_2_, 28.5% H_2_ and 5% N_2_, flow: 4.6 mL min^−1^) with *Clostridium ragsdalei*, a semi-continuous trickle bed reactor, consisting of a borosilicate glass column filled with 6 mm soda lime glass beads, reached an ethanol titer of 5.7 g L^−1^ [52].

**Table 8:** Application of cell immobilization for continuous solvent production with solventogenic and acetogenic clostridia

Strain	Immobilized system	Immobilization type and carrier	Substrate	Product	Max. productivity	References
*C. acetobutylicum* ATCC 55025	Continuous biofilm reactor	Adsorption, fibrous bed	Glucose and co-substrate butyrate	Butanol	4.6 g L^−1^ h^−1^	[114]
*C. acetobutylicum* B 5313	Two-stage chemostat with integrated solvent recovery	Adsorption, sugarcane bagasse	Glucose	ABE solvents	2.5 g L^−1^ h^−1^, 0.35 g g^−1^	[17]
*C. acetobutylicum* DSM 792	Continuous packet bed reactor	Adsorption, Tygon rings	Lactose and yeast extract (cheese whey imitate)	Butanol	4.4 g L^−1^ h^−1^	[207]
*C. acetobutylicum* DSM 792	Continuous biofilm reactor	Adsorption, coconut fibers and wood pulp	Sugar mix, synth. (lignocellulose hydrolysate)	ABE solvents	12.14 g L^−1^ h^−1^	[281]
*C. acetobutylicum* DSM792-ADH	Continuous packed bed reactor	Adsorption, wood pulp fibers	Lignocellulosic biomass hydrolysate	Isopropanol-butanol-ethanol mix	1.67 g L^−1^ h^−1^	[15]
*C. acetobutylicum* P-262	Continuous 4-stage biofilm reactor system	Adsorption, ceramic D-21 beads	Defibered-sweet-potato-slurry	ABE solvents	1.0 g L^−1^ h^−1^	[12]
*C. beijerinckii* BA101	Continuous packed bed reactor	Adsorption, clay bricks	Glucose	ABE solvents	15.8 g L^−1^ h^−1^	[240]
*C. beijerinckii* BA101	Continuous plug-flow biofilm reactor, in-site product recovery and effluent recycling	Adsorption, clay brick	Glucose	Butanol	16.2 g L^−1^ h^−1^	[168]
*C. beijerinckii* BA101	Continuous packed bed reactor, Scale-up	Adsorption, brick pieces	Glucose	Butanol	34.76 g L^−1^ h^−1^	[231]
*C. beijerinckii* DSM 6423	Continuous packed bed reactor	Adsorption, wood pulp	Sugar mix, synth. (lignocellulose hydrolysate)	ABE solvents	5.58 g L^−1^ h^−1^	[282]
*C. beijerinckii* NCIMB 8052	Chemostat with immobilized cells	Encapsulation, porous polyvinyl alcohol media	Glucose and co-substrate butyrate	Butanol	0.40 g L^−1^ h^−1^	[159]
*C. beijerinckii* ATCC 6014 and *C. tyrobutyricum* ATCC 25755	Continuous biofilm reactor	Adsorption, fibrous bed	Cassava bagasse hydrolysate	Isopropanol-butanol mix	0.44 g L^−1^ h^−1^	[336]
*C. carboxidivoran*s P7	Hollow fiber membrane biofilm reactor	Adsorption, membrane	Syngas: 20% CO, 5% H_2_, 15% CO_2_, 60% N_2_	Ethanol	23.93 g L^−1^, 0.24 mol C mol C^−1^	[267]
*C. carboxidivorans* P7	Biofilm reactor	Adsorption, cordierite-based ceramic monolith cylinder	Syngas: 20% CO, 5% H_2_, 15% CO_2_, 60% N_2_	Ethanol	4.89 g L^−1^, 2.35 g L^−1^ day^−1^	[266]
*C. pasteurianum* DSM 525	Continuous packed bed reactor	Adsorption, corn stover pieces	Glycerol	Butanol	4.2 g L^−1^ h^−1^	[86]
*C. ragsdalei* ATCC-PTA-7826	Trickle bed reactor semi-continuous	Adsorption, 6 mm soda lime glass beads	Syngas: 38% CO, 28.5% CO_2_, 28.5% H_2_, 5% N_2_	Ethanol	5.7 g L^−1^, 0.80 mmol L^−1^ h^−1^	[52]
*Clostridium ragsdalei ATCC-PTA-7826*	Trickle bed reactor semi-continuous	Adsorption, 6 mm soda lime glass beads	Syngas: 38% CO, 5% N_2_, 28.5% CO_2_, 28.5% H_2_	Ethanol	45 mg L^−1^ h^−1^	[53]

The overview is focused on the immobilized system, type and carrier

Regarding industrial use, there are patents for cell immobilization methods of solventogenic clostridia [44] and for gas fermentation with the acetogen *C. ljungdahlii* ERI2 ATCC 55380 for a 144-L trickle bed reactor [83]. For an overview of patents for biofilm reactors in gas fermentation, the reader is referred to Stoll et al. [275]. Nevertheless, maintenance of cell viability and physiology in an immobilized system is complicated [179]. Long-term biofilm stability is difficult to maintain and cell leakage from the support material requires an additional separation step [21, 225, 284]. Therefore, the scale-up of an immobilized system for industrial use is challenging and requires additional engineering studies [205, 231, 341]. In contrast, industrial gas fermentations using cell retention have already been demonstrated at scale. Due to the advantages of cell retention for process intensification, the number of applications for continuous high cell density fermentations is expected to increase significantly in the future.

### Integrated product recovery

Once the continuous cultivation for solvent production is established, the focus shifts to product toxicity of e.g. butanol or ethanol [133, 152]. One way to address product toxicity is to engineer solvent tolerant strains (see section “Strain engineering and design”).

A second approach is to reduce the concentration of toxic products in the fermentation broth by integrating solvent recovery into the upstream process by in situ or in-line methods [58, 82, 161]. The in-line method is maintained in a separate loop, circling the alcohol-depleted effluent back into the reactor, whereas the culture broth does not leave the reactor during in situ product recovery (see Fig. 2g, h) [82, 306]. The spatial separation of in-line recovery methods from the fermentation process allows independent optimization [82]. The constant recovery of toxic products lowers the actual concentration in the culture broth [21]. That way, product inhibition is decreased, leading to increased solvent yields and productivities and improved substrate conversion rates [164, 273, 325, 328].

The traditional method for product recovery is by distillation using multi-column procedures, particularly in the industrial production of fuel ethanol [21, 306, 327]. The growing interest of the biofuel industry to use lignocellulosic and waste stream feedstocks leads to lowered alcohol concentrations in the fermentation broth [306]. Distillation is a robust and popular method for ethanol recovery but is less suitable for low solvent concentrations due to the high energy requirement [21, 306, 327]. The required energy for the distillation procedure increases exponentially for butanol levels below 10 g L^−1^ [190] or ethanol concentrations below 40 g L^−1^ [306]. The boiling point of butanol is higher than that of water. These azeotropic properties hinder the butanol recovery via distillation [117].

Requirements concerning the degree of recovery differ between integrated methods and methods for the final separation at the end of the bioprocess. While the effluent during integrated recovery is circulated by feeding back to the reactor, the product remaining in the effluent after the recovery with final separation technologies like distillation is lost. Consequently, final separation technologies require a higher degree of alcohol recovery [82, 161, 306].

Therefore, alternative methods are employed for integrated product recovery, including gas stripping, liquid–liquid extraction, adsorption, pervaporation and perstraction [65, 82, 166]. Below, we give a short introduction to recovery techniques. For further information, the reader is pointed out to Friedl [82], Vane [306], Bharathiraja et al. [21] and Kujawska et al. [152].

*Gas stripping* is a simple, physical method for economic in situ solvent recovery [55, 82]. For separation, the cell broth in the reactor is flushed with N_2_ or CO_2_, stripping the volatile solvents from the solution [21]. Afterwards, the stripped solvents and entrained water is recovered by condensation from the escaping gas stream [21, 55, 74]. The gas stream can be recycled for several cycles [66]. To lower processing costs, there is also the possibility to directly use the fermentation gas (containing CO_2_ or H_2_) as stripping gas [82]. Gas stripping is a quite flexible separation method and can be used in combination with different process types (e.g. batch, fed-batch, continuous, multi-stage processes, fluidized bed reactors) and with other separation techniques [21, 73, 82]. It is claimed as the most studied technique for solvent recovery and as one of the most energy efficient and economic methods [101, 166, 228]. Ezeji et al. [69, 70] showed that gas-stripping efficiently lowers the solvent concentration in the reactor, leading to a 200% improved solvent productivity and 118% improved yield. Friedl [82] suggested the in-line recovery for gas stripping in an external loop as it offers easier optimization of the recovery rate compared to in situ recovery.

*Liquid–liquid extraction* recovery is realized using an extracting solvent showing a miscibility gap with water and high affinity to the product [82]. The advantages of this method are high capacity and selectivity. However, the design of the extraction process can be complex and expensive to perform [100, 161]. Implementation of in situ liquid–liquid extraction requires a non-toxic extraction solvent [58]. The most recommended non-toxic extraction solvent for in situ recovery in an ABE fermentation is oleyl alcohol [17, 60, 233, 283, 339]. The currently known extraction solvents are applicable, but not ideal in performance, making the choice of the extraction solvent a challenging and ongoing research topic [82].

Of the *membrane techniques* for solvent recovery, *perstraction and pervaporation* are the two most promising ones. *Perstraction* is an expansion of liquid–liquid extraction. The separation of cell broth and extracting solvent via a suitable membrane eliminates the problem of extraction of solvent toxicity and emulsion development [58, 82].

*Pervaporation* or so called “membrane distillation”, is claimed to be commercially competitive and the best-developed method for in situ solvent removal [21, 82, 121, 226, 305]. Hydrophobic polymeric membranes allow solvents to selectively permeate from the liquid fermentation broth on one membrane site into the gas phase on the other membrane site [305]. The membranes possess a higher affinity to organic solvents, leading to high fluxes and a fast sorption of the organic compounds [305]. The driving force of pervaporation is the difference of vapor pressure between the feed and permeate side [21, 82, 305]. The difference is typically introduced by the application of a vacuum or sweep gas on the permeate side of the membrane [305]. PDMS (polydimethylsiloxane [121, 305], and POMS (polyoctylmethylsilixane [156], are typical used polymers for the pervaporation membranes. For more information on the membrane material, the reader is pointed to Huang et al. [111].

For the in situ implementation of membrane techniques, the membranes need to be mounted inside the reactor. While successfully implemented in lab-scale, the design and scale-up are quite complicated [82]. Common problems of membranes such as fouling and clogging can lead to operational problems since there is no possibility for cleaning when used in situ [58, 82]. The disadvantages of membrane techniques such as high price, limitation of diffusion and fouling problems constitute an obstacle for the implementation of perstraction and pervaporation on an industrial scale [58, 166].

*Adsorption* is an effective, energy-efficient, and easy to operate separation technique [64, 82, 230]. It has been investigated in several process mode combinations and showed to reduce the inhibiting product concentration [99, 175, 214, 215, 254, 329, 330]. The solvent recovery by adsorption of the fermentation broth can be operated continuously and is carried out in two steps: First, the alcohol is taken up by the adsorbent until maximum loading is obtained. Subsequently, the adsorbent is regenerated to obtain a concentrated butanol solution [306, 325]. Regeneration is accomplished by temperature increase or by reduction of the pressure [82]. For continuous mode, more than one column with adsorption material is needed [82]. Depending on the material, adsorption offers the possibility for selective removal of solvents in a gaseous, vapor or liquid mixture and can also be used with other separation methods to reduce the water content of the concentrated product [82]. Typical adsorption materials are hydrophobic activated carbon, zeolites and polymeric (ion-exchange) resins [82, 111, 325]. According to Abdehagh et al. [1], activated carbon F-400 is the best butanol adsorbent with the highest adsorption capacity, while Friedl [82] pointed out that zeolites are already successfully used in industrial plants for ethanol dehydration. A disadvantage of adsorption for the integration into a fermentation process is the problem of nutrient fouling, which requires the pre-separation with micro- or ultrafiltration before recovering the solvents by adsorption [82]. Depending on the material, adsorption suffers from low selectivity, high resin prices and physical instability [58, 166]. Therefore, the performance of adsorption needs to be evaluated on an industrial scale [82].

Each integrated product recovery method has its benefits and drawbacks [166]. The main target for implementation in the solvent producing industry is the energy-efficiency of the separation method. To minimize the costs and increase the productivity, the recovery step needs to be operable in continuous mode without interferences.

While especially required in fed-batch processes where product inhibition is limiting the productivity, these integrated product recovery strategies have also been applied to continuous processes and systems using cell immobilization [30, 70, 73, 100, 182, 325, 330].

Most frequently used recovery methods in lab-scale are gas stripping and pervaporation, implemented in several investigations [31, 73, 74, 162, 182, 226, 248, 251, 268, 305]. For example, Lienhardt et al. [168] used a continuous biofilm reactor with *Clostridium beijerinckii* BA101 cells adsorbed onto clay bricks fed with glucose as substrate. The reactor effluent was recycled after the removal of butanol by pervaporation, lowering butanol toxicity while retaining the intermediate acids in the effluent. At a dilution rate of 2.0 h^−1^, Lienhardt et al. [168] obtained complete sugar utilization with a productivity of 10.2 g^−1^ L^−1^ h^−1^. In industry, combinations of two recovery methods are also applicable, e.g. the combination of liquid–liquid extraction of butanol with oleyl alcohol coupled with gas stripping (patented by Butamax Advanced Biofuels LLC [93]).

One example for successful application of in situ product recovery to increase titer, rate and yield metrics in a continuous fermentation process relied on pervaporation and showed a significant increase of the substrate consumption rate, solvent productivity, and yield by 58% (2.02 g L^−1^ h^−1^), 81% (0.75 g L^−1^ h^−1^) and 15% (0.38 g g^−1^), respectively [162]. Using cassava-derived glucose with *Clostridium acetobutylicum* DP217, final ABE and butanol titers of 574.3 g L^−1^ and 501.1 g L^−1^, respectively, were obtained [162]. In a recent study, systems biology tools enabled the engineering of *C. acetobutylicum* and achieved a stable, highly selective, and high yield butanol production of 0.35 g g^−1^, which corresponds to 84% of the theoretical maximum [210]. Using the strain in a continuous high cell density cultivation combined with in situ product recovery, a butanol titer of 550 g L^−1^ was achieved in the recovered product stream, comparable to solvent levels in traditional ethanol plants [210]. The implementation of an integrated product recovery method into an optimized continuous process has therefore been shown to increase the final titer and consequently the economic competitiveness for industrial production.

## Industrial application

A look in the past shows the development of industrial applications of solvent production with clostridia. In the 1970s the oil crisis led to a revival of the historical Weizmann process, which was initially established in 1915 during the First World War but with rising substrate prices of molasses, maize, or wheat the ABE process was no longer economically viable [58, 312]. In 2006, DuPont and British Petroleum (BP) announced their cooperation for the reinstallation of new industrial ABE plants, once again leading to an increased research interest in the topic of ABE processing [312]. At the same time, gas fermentation technology using acetogens received increasing attention and has been developed towards industrial implementation. Since then, many plants and projects launched the production of butanol, acetone, and ethanol with solventogenic and acetogenic clostridia from various feedstocks but several needed modernizations or were closed due to economic pressure [211, 326]. In the following, an overview of the recent companies in the field of ABE fermentation and gas fermentation is shown.

### Traditional ABE fermentation

Some of the major companies for the industrial ABE process with solventogenic clostridia are Butamax Advanced Biofuels, ButylFuel LLC, Celtic Renewables Ltd and Cathay Industrial Biotech. Companies working on the process development for biobutanol production are Tetravitae Bioscience and METabolic EXplorer [2].

*Butamax Advanced Biofuels (US)*, a joint venture of BP and DuPont, currently operates a biobutanol plant in Lamberton, Minnesota with a capacity to produce 30,000 tons butanol annually from lignocellulosic feedstocks. In addition, Butamax operates a demonstration facility in Hull (UK) and a small-scale unit in Delaware (US), which is using corn and sugar as feedstock. Since no details of the process have been released, it was assumed that Butamax relies on a traditional process using *C. beijerinckii*.[2, 29, 58, 211]

*ButylFuel LLC (US)* created a patented two-stage fermentation process with *C. tyrobutyricum* and *C. acetobutylicum* (see section “Multi-stage systems”) and uses forest residues, temperate grasses and crop residuals as feedstock for butanol production [58, 244].

*Celtic Renewables Ltd (UK)* was formed in 2012. In 2017, the company announced the construction of a commercial demonstration plant in Grangemouth, UK for over 500,000 L of biofuel annually. Originally developed and established in 2007 at the Edinburgh Napier University, the ABE process of Celtic Renewables uses a *Clostridium* sp. able to convert xylose, arabinose and glucose into butanol, ethanol, and acetone. As feedstocks, the whisky by-products pot ale and draff are used, pretreated by thermal hydrolysis. Additionally, solid residues and cell biomass generated during the ABE process is sold as high-grade animal feed [34].

Two former biobutanol companies are *Green Biologics Ltd.* (UK) and *Cobalt Technologies Biofuels* (USA). Cobalt Technologies has been operating a pilot plant production for 20,000 L of butanol annually until bankruptcy in 2015 [2, 155]. Green Biologics Ltd. developed a new fermentation process with genetically modified strains and started a commercial butanol production in 2017, aiming to produce from sugar and agricultural waste. Unfortunately, they ran out of money in October 2019 and now offer their knowledge under the name ‘Biocleave Limited’ [24, 94].

### Gas fermentation

Three companies are known for their gas fermentation technology and pilot plants: INEOS Bio, Coskata Inc. and LanzaTech. However, only LanzaTech prevailed and has now implemented its technology in a commercial plant at scale [275].

*Coskata Inc.* was originally founded in 2006 in cooperation with the University of Oklahoma. For the process, methane was reformed into syngas and fermented to ethanol, presumably using HFMBR technology [107, 275]. Coskata was operating a gas fermentation plant for ethanol production from 2009 to 2011 (capacity of 118 t a^–1^) in Pennsylvania, which was shut down due to financial insolvency in 2015. The Coskata technology was acquired by Synata Bio in 2016 [107, 275].

*INEOS Bio* was established in 2008, because of the takeover by Bioengineering Resources Inc., which was founded by the gas fermentation pioneer James L. Gaddy [11, 170, 275]. INEOS Bio was one of the first companies, implementing gas fermentation at an industrial scale [149, 275]. In 2012 they started operating a semi-commercial biorefinery plant in Florida, aiming a bioethanol production of 8 million gallons per year (Mgy), produced from syngas, generated from lignocellulosic biomass and municipal waste [241, 275]. The presence of the inhibitor hydrogen cyanide in the feed gas led to severe problems with the syngas fermentation process in 2013. In consequence, the plant was shut down in December 2014 [107, 275]. In 2017 the plant was sold to Alliance Bio-Products Inc. During the same time, INEOS Bio was purchased by *Jupeng Bio, Inc.* (Texas, US). Jupeng Bio claims to be the first company worldwide introducing large scale cellulosic bioethanol in 2013. For their syngas fermentation they gasify mainly biomass material and waste material [136].

*LanzaTech* based in Illinois (US) was founded by the gas fermentation pioneers Sean D. Simpson and Richard Forster in 2005 [275]. As one of the first, LanzaTech managed to establish a profitable, stable, and continuous gas fermentation process using syngas for selective ethanol production [107, 108, 147, 149, 275]. Their process was initially extensively tested in a pilot plant in Glenbrook, New Zealand, using steel mill exhaust gases for ethanol production with a proprietary *C. autoethanogenum* strain [48, 108]. LanzaTech set up two gas fermentation demonstration plants in cooperation with the large Chinese steel manufacturers BaoSteel and Shougang (capacity 300 Mt a^–1^ ethanol) and now aims to construct numerous commercial plants worldwide [107, 275]. In cooperation with Aemetis, LanzaTech build a plant for biogenic syngas in California, targeting the gasification of non-recyclable MSW, agricultural and forestry waste [139, 149, 275]. With their strong international network, LanzaTech continues to advance gas fermentation technology and to expand to additional products [275]. In addition, LanzaTech has a broad patent portfolio to secure its intellectual property in process technology and strain development and is ready to implement production of chemicals such as 2,3-butanediol, butanol, butadiene and acetone in commercial plants [149, 275].

## Prospects

In this review, we show that the intelligent connection of bioprocess technology and strain engineering tools complemented by newly gained knowledge from systems biology studies is the ideal way towards highly efficient fermentation processes for industrial solvent production, competitive to non-sustainable fuel and solvent industry [210]. Nevertheless, the industrial implementation of the continuous fermentation process is limited to one example, the LanzaTech process using CO from steel mill off the gas to produce ethanol with *C. autoethanogenum*.

The development of solutions providing high productivities will be an impetus to establish continuous bioprocesses for economic solvents and bulk chemical production [183]. Moreover, the utilization of alternative low-cost feedstocks such as lignocellulosic and gaseous substrates will increase economic viability. The progress in the understanding and design of strains as well as fermentation strategies with high cell density cultivation using cell retention techniques and the integration of in situ product recovery methods shows an enormous potential for continuous fermentations.

To exploit this potential, a detailed understanding of strain physiology and metabolism under “production conditions” is required. Using systems level analyses and metabolic modeling, targets for strain improvement can be identified, and emerging genome engineering tools allow to rapidly establish phenotype-genotype relationships.

Additionally, the transfer of promising bioprocessing concepts into larger scales requires the development and implementation of process analytical technology (PAT) concepts to obtain suitable monitoring and control strategies. Here, methods like flow cytometry emerge as promising tools to monitor active biomass in “dirty” substrates, e.g. lignocellulose-based hydrolysates containing particles, thus allowing to obtain more solid data in terms of process performance. Furthermore, flow cytometry can increase knowledge on sporulation and cell viability under different conditions. Other process analyzers include spectroscopy methods, but also omics tools to assess culture response to inhibitors and varying feedstock compositions. Finally, the demand for enhanced control promotes the development of models, ranging from simple software sensors and black box models to fully integrated spatiotemporal models.

Following this integrated approach shall ultimately allow to successfully scale-up and implement novel continuous bioprocessing solutions for solvent production or altogether new products using solventogenic and acetogenic clostridia.
